# Role of a functional polymorphism in the *F2R* gene promoter in sarcoidosis

**DOI:** 10.1111/resp.12608

**Published:** 2015-08-17

**Authors:** Manuela Platé, Phillippa J. Lawson, Michael R. Hill, Richard P. Marshall, Helen L. Booth, Meena Kumari, Geoffrey J. Laurent, John R. Hurst, Rachel C. Chambers

**Affiliations:** ^1^ Centre for Inflammation and Tissue RepairUniversity College LondonLondonUK; ^2^ Department of Thoracic MedicineUniversity College HospitalLondonUK; ^3^ Faculty of Population Health SciencesUniversity College LondonLondonUK

**Keywords:** F2R promoter, proteinase‐activated receptor‐1, sarcoidosis, single nucleotide polymorphism

## Abstract

Sarcoidosis is a multisystem granulomatous disease of unknown aetiology characterized by increased inflammation, and results from gene–environment interactions. Proteinase‐activated receptor‐1 mediates the interplay between coagulation and inflammation. The rs2227744G > A promoter single nucleotide polymorphism has been linked to inflammation, cardiovascular disease and chronic obstructive pulmonary disease exacerbations. Using a case‐control study (184 cases with sarcoidosis and 368 controls), we show that the rs2227744A allele significantly associates with protection from sarcoidosis (*P* = 0.003, OR = 0.68 (0.52–0.88)).

**Abbreviations**
CI: confidence interval
COPD: chronic obstructive pulmonary disease
ELSA: English longitudinal study of ageing
F2R: factor 2 receptor
FEV_1_: forced expiratory volume in 1 s
FVC: forced vital capacity
HLA: human leukocyte antigen
HWE: Hardy–Weinberg equilibrium
MHC: major histocompatibility complex
OR: odds ratio
PAR‐1: proteinase‐activated receptor‐1
SNP: single nucleotide polymorphism

Sarcoidosis is a multisystem granulomatous disease of unknown aetiology.^1^ The manifestations of the disease are diverse,^1^ but the lungs are affected in 95% of patients^2^ with an inflammatory response characterized by the development of non‐caseating granuloma.^3^ The phenotype varies in severity with a key distinction being the presence or absence of persistent disease. Up to 17% of UK sarcoidosis patients will develop pulmonary fibrosis.^4^ The disease tracks in families suggesting genetic predisposition which has been most closely related to the major histocompatibility complex (MHC) region on chromosome 6. This region includes the human leukocyte antigen (HLA)‐class I and class‐II genes and a large number of genes involved in immune and inflammatory responses.^5^ Moreover, there is evidence of increased procoagulant activity in sarcoidosis, which significantly correlates with lung function.^6^

Activation of proteinase‐activated receptor‐1 (PAR‐1) by coagulation proteinases contributes to lung inflammation and fibrosis, and we have shown that PAR‐1 knock‐out mice are protected when these conditions are experimentally induced.^7^, ^8^ The minor allele of the rs2227744G > A single nucleotide polymorphism (SNP) in the promoter of the factor 2 receptor (*F2R*) gene (chromosome 5q13.3) has been demonstrated to confer higher promoter activity, and hence higher PAR‐1 expression levels, *in vitro.* 9 The same SNP has been shown to be associated with protection from frequent chronic obstructive pulmonary disease (COPD) exacerbations^9^ and with inflammation and cardiovascular disease.^10^

A UK Caucasian White population of 184 sarcoidosis subjects recruited from University College London Hospital, the Royal Free Hospital and St George's Hospital was used for this study.^11^ Sarcoidosis was diagnosed according to international guidelines^1^ and patients were sub‐classified into those with persistent and non‐persistent disease.^11^ Three hundred sixty‐eight age and gender‐matched UK Caucasian White controls were selected among the National Blood Service individuals, Whitehall II and English longitudinal study of ageing (ELSA) cohorts^12^, ^13^ (Table 1). Individuals with lung disease in these latter cohorts were excluded for the purpose of this study. All aspects of the study had ethics committee approval, and written informed consent was obtained from all participants.

**Table 1:** Clinical characteristics of the sarcoidosis patients and healthy controls

Characteristics	Sarcoidosis (*n* = 184)				Controls (*n* = 368)
	Non‐persistent (*n* = 97)	Persistent (*n* = 71)	*P*‐value (non‐persistent vs persistent)	All patients	All controls
Age (years)	38.7 (10.8)	40.7 (12.1)	0.27	39.7 (11.4)	40.7 (10.4)
Male	37 (38%)	32 (45%)	0.08	75 (41%)	149 (41%)
FEV_1_ (L)	2.90 (0.89)	2.33 (0.88)	<0.001	2.67 (0.92)	NA
FVC (L)	3.62 (1.03)	3.19 (1.10)	0.024	3.44 (1.06)	NA
FEV_1_/FVC	0.80 (0.09)	0.73 (0.12)	<0.001	0.77 (0.11)	NA

Data are presented as mean (standard deviation) or number (%). FEV_1_, forced expiratory volume in 1 s; FVC, forced vital capacity; NA, not available. Total number of sarcoidosis patients includes 16 unclassified patients.

Genotyping of the Whitehall II and ELSA cohorts was performed by KBiosciences using the KASPar assay (LGC, Middlesex, UK), based on Kompetitive Allele Specific PCR or KASP technology. For the sarcoidosis cohort and National Blood Service samples, a region containing the rs2227744G > A polymorphism was PCR amplified and the products sequenced using the MegaBACE automated sequencing and a MegaBACE 1000 DNA analysis system (GE Healthcare, Little Chalfont, UK) following the manufacturer instructions. Samples were analysed using a MegaBACE 1000 DNA analysis system.

For phenotypic data, categorical data were tested for differences between cases with persistent and non‐persistent disease by means of a chi‐square test, and continuous data were tested using the 2‐tailed Student *t*‐test. For genotypic data, all procedures were performed using PLINK v1.07.^14^ Hardy–Weinberg equilibrium (HWE) was tested using the exact test described by Wigginton^15^ and implemented in the PLINK software.^14^ For each analysed group, standard case‐control analyses on allele and genotype frequency data were performed with logistic regression with correction for age and gender. The analyses were also repeated assuming a dominant and a recessive model of inheritance.

The characteristics of the sarcoidosis patients and healthy controls are reported in Table 1. Lung function data are not available for the healthy blood donors. Patients with persistent sarcoidosis have poorer lung function and a restrictive forced expiratory volume in 1 s/forced vital capacity (FEV_1_/FVC) ratio of 70% or greater as would be expected.

A large cohort of 8579 UK Caucasian healthy individuals, who participated in the ELSA or Whitehall II study, was previously genotyped for this polymorphism.^9^ The minor allele frequency in the controls selected for this study (0.43) was comparable to that observed in this population (0.45)^9^ and to those found in publicly available database for European cohorts (HapMap3: 0.43 and ALFRED: The Allele Frequency Database: 0.42). The genotype distributions in the ELSA and Whitehall II cohorts were calculated (AA/AG/GG 0.20/0.50/0.30) and were very similar to those observed in our control population (0.20/0.46/0.34) and to those reported in the HapMap3 database (0.18/0.50/0.32).

No significant deviation from HWE was observed in cases (*P*‐value = 0.62) or controls (*P*‐value = 0.34) or both (*P*‐value = 0.21). Association analyses showed that the rs2227744A minor allele is associated with protection from sarcoidosis but not with persistent and non‐persistent phenotype. Allelic chi‐square analyses of cases and controls showed that the rs2227744A allele was significantly less frequent in cases compared with controls, with the additive model being the most significant (*P*‐value = 0.003, OR = 0.68 (0.52–0.88)). The association was confirmed by genotypic analyses (*P*‐value = 0.01) (Table 2). Comparing the allelic frequencies in the persistent and non‐persistent disease groups, no significant association was found at the allelic (*P*‐value = 0.57, OR = 1.2 (0.64–2.27) or genotypic level (*P*‐value = 0.34) (Table 2).

**Table 2:** Allelic and genotypic association analyses with sarcoidosis

	Groups	Test	Cases (freq)	Controls (freq)	*P*‐value	OR (95% CI)
Cases versus controls	A/G	Allelic	0.33/0.67	0.43/0.57	**0.003**	0.63 (0.52–0.88)
		Dominant			**0.008**	0.61 (0.42–0.88)
		Recessive			**0.03**	0.57 (0.34–0.99)
	AA/AG/GG	Genotypic	0.12/0.43/0.45	0.20/0.46/0.34	**0.01**	NA
Persistent versus non‐persistent	A/G	Allelic	0.28/0.72	0.345/0.655	0.57	1.2 (0.64–2.27)
		Dominant			0.77	1.13 (0.50–2.53)
		Recessive			0.42	1.9 (0.38–10)
	AA/AG/GG	Genotypic	0.10/0.37/0.53	0.11/0.47/0.42	0.34	NA

CI, confidence interval; Freq, frequency; NA, not applicable; OR, odds ratio. Significant results are indicated in bold.

Sarcoidosis is believed to result from gene–environment interactions, though the nature of the environmental stimulus remains obscure. Multiple genome‐wide association study and candidate gene studies have suggested relationships between loci and the development, phenotype or clinical course of sarcoidosis most often in relation to the MHC/HLA genes on chromosome 6.^5^ The locus 5q13.3 containing the SNP has not so far been identified as associated with sarcoidosis. We found that the rare rs2227744A PAR‐1 allele is less common in subjects with sarcoidosis than in controls, but that this did not track with the key persistent/non‐persistent clinical phenotype. Since the rs2227744A allele in functional studies is associated with increased *F2R* promoter activity, it is tempting to speculate that an enhanced PAR‐1 signalling response afforded by increased PAR‐1 expression may, in part, be responsible for protection from the development of sarcoidosis. This is consistent with our finding that the same allele is also protective towards frequent exacerbations in COPD.^9^ The rs2227744A allele does not distinguish between persistent and non‐persistent disease, suggesting that this allele protects from the development of sarcoidosis but does not influence the course of the disease. We therefore speculate that the effect of PAR‐1 in sarcoidosis may be related to influences on the early inflammatory stage of the disease. However, the lack of statistical significance for the comparison between persistent and non‐persistent phenotype might reflect a lack of statistical power, given the small sample size associated with the sub‐group analysis (power 14.3%). Finally, it is also worth mentioning that our study did not include a replication cohort so that our observations require further confirmation.

## Acknowledgements

We thank all participating Civil Service departments and their welfare, personnel and establishment officers; the Occupational Health and Safety Agency; the Council of Civil Service Unions; all participating civil servants in the Whitehall II study; and all members of the Whitehall II study team. We wish to thank the National Centre for Social Research (NCSR) who collected the ELSA samples used in this study and all the participants. We thank Dr Huw Beynon and Dr Paul Dilworth (Royal Free Hospital, London), Dr Charlotte Rayner (St George's Hospital, London) and Kevin Hart (National Blood Service, London) for providing access to sarcoidosis patients and controls. We are also very grateful to Cathy Read who collected blood samples at the UCL Hospitals. We would finally like to thank all the patients participating in the Sarcoidosis cohort. We also wish to thank Dr Ilaria Guella (UBC) for helpful discussion on data analysis. This work was supported by a British Lung Foundation, Garfield Weston Foundation award (Grant reference COPD10‐6), a Wellcome Trust Clinical Training Fellowship (074558) and a Wellcome Trust program grant (051154). The Whitehall II study has been supported by grants from the Medical Research Council (MRC); Economic and Social Research Council; British Heart Foundation (RG/02/005; PG/03/029); Health and Safety Executive; Department of Health; National Heart Lung and Blood Institute (HL36310); US NIH National Institute on Aging (AG13196); US NIH Agency for Health Care Policy Research (HS06516); and the John D. and Catherine T. MacArthur Foundation Research Networks on Successful Midlife Development and Socioeconomic Status and Health. The US National Institute on Aging (grant numbers R01AG24233, R01AG1764406S1) funds the English Longitudinal Study of Ageing DNA Repository (EDNAR), and ELSA is funded by the National Institute of Aging in the USA and a consortium of UK Government departments coordinated by the Office for National Statistics. We declare that the funders of this project played no role in the design of the study, the collection and analysis of the data, or in the preparation of the manuscript.
